# Online Running-Gait Generation for Bipedal Robots with Smooth State Switching and Accurate Speed Tracking

**DOI:** 10.3390/biomimetics8010114

**Published:** 2023-03-10

**Authors:** Xiang Meng, Zhangguo Yu, Xuechao Chen, Zelin Huang, Chencheng Dong, Fei Meng

**Affiliations:** 1 School of Mechatronical Engineering, Beijing Institute of Technology, Beijing 100081, China; 2 Key Laboratory of Biomimetic Robots and Systems, Ministry of Education, Beijing 100081, China; 3 Beijing Advanced Innovation Center for Intelligent Robotics and Systems, Beijing Institute of Technology, Beijing 100081, China

**Keywords:** bipedal robots, online running-gait generation, state switching, speed tracking

## Abstract

Smooth state switching and accurate speed tracking are important for the stability and reactivity of bipedal robots when running. However, previous studies have rarely been able to synthesize these two capabilities online. In this paper, we present an online running-gait generator for bipedal robots that allows for smooth state switching and accurate speed tracking. Considering a fluctuating height nature and computational expediency, the robot is represented by a simplified variable-height inverted-pendulum (VHIP) model. In order to achieve smooth state switching at the beginning and end of running, a segmented zero moment point (ZMP) trajectory optimization is proposed to automatically provide a feasible and smooth center-of-mass (CoM) trajectory that enables the robot to stably start or stop running at the given speed. To accurately track online the desired speed during running, we propose an iterative algorithm to compute target footholds, which allows for the robot to follow the interactive desired speed after the next two steps. Lastly, a numerical experiment and the simulation of online variable speed running were performed with position-controlled bipedal robot BHR7P, and the results verified the effectiveness of the proposed methods.

## 1. Introduction

Legged robots, as a typical category of bionic robots, have significantly developed in recent years [1,2,3,4,5,6,7,8]. With a humanlike appearance, bipedal robots can replace humans to complete some repetitive tasks without changing the work environment. Most bipedal robots are able to walk stably at a relatively low speed [1,2,3]. In [1], Kajita et al. proposed the preview control of a zero moment point to generate a walking pattern for bipedal robot HRP2. Kuindersma et al. [2] adopted a convex optimization involving whole-body control to achieve stable walking on unstructured terrains with humanoid robot Atlas. In [3], Chen et al. proposed a bioinspired walking control strategy to enable bipedal robot BHR5 to walk stably with toe-off, heel-strike, and disturbance rejection. However, these methods were designed for a walking motion at a relatively slow speed, which restricts the agility of bipedal robots. Running is a kind of motion that can significantly improve the moving speed of bipedal robots, but the stability and reactivity of bipedal running are still challenging [9,10,11,12].

On the one hand, different from walking, bipedal running requires the fluctuation of the height of the robot’s center of mass (CoM) to provide sufficient contact force entering the flight phase. Therefore, the widely used linear inverted-pendulum (LIP) model with fixed height is not applicable. Nevertheless, the nonlinearity and time-varying nature of the dynamic model results in increased computational complexity, hindering online motion planning. On the other hand, when the robot is running, smooth state switching and accurate speed tracking are crucial for its stability and reactivity. Unresolved problems are feasible motion planning during state switches to ensure that the robot stably starts or stops running at the desired speed, and the computation of appropriate footholds to accurately track the commanded speed online.

There are some impressive running-gait generators for bipedal robots using the inverted-pendulum model and its variants [9,10,11,12], including verification on real hardware. These approaches typically substitute the contact force with the zero moment point (ZMP) [13], and the robot is reduced to an inverted-pendulum model. In [9], an offline running motion planning was implemented on a bipedal robot where the CoM trajectory in state switching was available through the craft interpolation and the footholds during running were predefined. A similar approach was adopted in [10] to enable the running-gait generation for a bipedal robot with 7 redundant degrees of freedom in each leg. In [11,12], the divergent component of the reduced-order model was used to plan the running motion, but the study focused on the generation of a cyclic gait pattern when the footholds were provided in advance. In these approaches, the craft interpolation of the CoM trajectory during state switching is tedious and it forces the robot to start or stop running with marching in place. The robot’s running speed is determined by the predefined footholds and step timings, which restricts the reactivity of the robot.

It has become popular to plan dynamic motions for bipedal robots [14,15,16,17,18] using the centroidal dynamics model [19]. This kind of method commonly considers the dynamics between the contact force and the centroidal momentum by formulating a nonlinear trajectory optimization (TO) problem to optimize the centroidal momentum trajectory and the variation in the contact force during running. In [14], a periodic whole-body running movement for a humanoid robot, Atlas, is optimized with a single TO involving centroidal dynamics and the full-body kinematics model. Even though optimized running motion using these methods is feasible and natural, it tends to require solving multivariate nonlinear programming (NLP) problems, and its computational complexity prevents it from online implementation. Those techniques that account for the full-rigid-body dynamics model for running-gait generation [20,21,22] suffer from a similar dilemma. Due to high-dimensional nonlinearity, a full-body running motion within a limited time horizon often takes minutes or even hours to be optimized offline.

The capturability constraint is broadly used in bipedal locomotion control [23,24]. By employing the constraint, the footholds of the next N steps can be computed to prevent the robot from falling. Similarly, it can be utilized to calculate appropriate footholds to control the speed of the robot. In [25], a dynamic running point-footed bipedal robot driven by hydraulic and pneumatic actuators realized online speed adjustment through heuristic foothold control. However, these methods mainly constrained the capturability of the CoM position to control the robot back to a stable motion state, rarely concentrating on the state switching and quantitative tracking of the desired speed.

In this paper, to enhance the stability and reactivity of bipedal robots, we propose an online running-gait generator that could realize smooth state switching and accurately track the desired speed online. For online computational expediency, the bipedal robot is represented by a simplified variable-height inverted-pendulum (VHIP) model [26]. For smooth motion-state switching at the beginning and end of running, a segmented ZMP trajectory optimization is proposed to allow for the smooth transition of the CoM trajectory. During bipedal running, we did not predefine the desired footholds, but provided the nominal footholds for the generator. Combined with the capturability constraint of the CoM velocity, the target footholds were computed using an iterative algorithm to accurately track the desired speed online.

The main contributions of this paper are as follows:

- An online running-gait generator for bipedal robots with a VHIP model is proposed that can generate complete running motion with the smooth state switching and accurate tracking of the desired speed.
- Segmented ZMP trajectory optimization is formulated at the beginning and end of the running, which could automatically provide a feasible and smooth CoM trajectory during state switching. This allows for the robot to start or stop running stably at the given speed.
- We designed an iterative speed-guided foothold algorithm with the capturability constraint of CoM velocity during running that could accurately track the desired speed in the next two steps.

This paper is structured as follows. In Section 2, we describe the running-gait generation with a simplified VHIP model. In Section 3, we elaborate on the proposed segmented ZMP trajectory optimization for smooth state switching and the online iteration algorithm of target footholds to realize accurate following of the desired speed. In Section 4, the simulation results of the complete running motion planning for position-controlled bipedal robot BHR7P using the proposed generator are presented. Lastly, the conclusion and future work are stated in Section 5.

## 2. Running-Gait Generation with a Simplified VHIP Model

In this section, we analyze the VHIP model and its dynamics. Considering the time-varying dynamics and online computational expediency, running-gait generation was split into two sequential parts: vertical CoM generation and horizontal CoM computation. Using the Euler method, the time-varying dynamics equations could be written as tridiagonal linear equations with the planned CoM height trajectory. On the basis of the linear equations, the horizontal CoM trajectory could be computed efficiently with the capturability constraint of the CoM velocity.

### 2.1. Variable-Height Inverted-Pendulum Model

**Figure 1:**
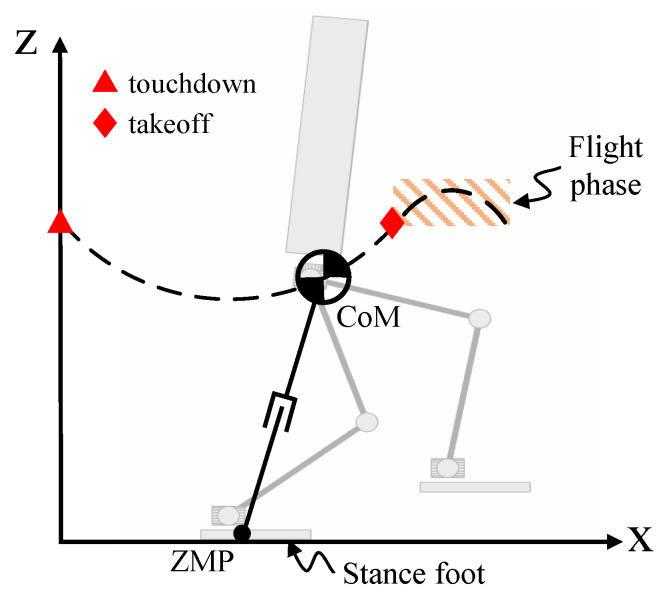
Variable-height inverted-pendulum (VHIP) model.

As shown in Figure 1, the bipedal robot was simplified into a VHIP model. Under the assumption that the robot was running on flat ground, it was possible to replace the contact force with ZMP in the stance phase. When the robot entered the flight phase, it moved the parabola because there was no external contact force affecting it except for gravity.

Therefore, we focused on the dynamics of the VHIP model in the stance phase. The dynamics equations are as follows:

(1) $$p^{x,y}=c^{x,y}-\frac{c^{z}}{{\ddot{c}}^{z}+g}{\ddot{c}}^{x,y}+S\frac{{\dot{L}}^{x,y}}{m({\ddot{c}}^{z}+g)},\;S=\left[\begin{array}{cc} 0 & -1\\ 1 & 0 \end{array}\right]$$

where p and c are the position of ZMP and CoM in the global frame, respectively, x,y,z represents the quantity on the corresponding axis, *m* is the total mass of the robot, *g* is the acceleration of gravity, $\dot{L}$ is the rate of the centroidal angular momentum, and S is a simple rotational matrix.

Under the assumption that the rate of centroidal angular momentum was relatively small compared with the position and acceleration of CoM, we ignored the third term on the right-hand side of (1), i.e., $\dot{L}=0$, so (1) could be simplified in the following way:

(2) $$p^{x,y}=c^{x,y}-\frac{c^{z}}{{\ddot{c}}^{z}+g}{\ddot{c}}^{x,y}$$

In (2), the height of the CoM $c^{z}$ and its acceleration ${\ddot{c}}^{z}$ are time-varying quantities. It is difficult to simultaneously plan the feasible trajectories of a three-dimensional CoM. In order to simplify CoM planning, the vertical height was extracted under the assumption that it was independent of the horizontal movement. In this way, the height of CoM could be generated as a known quantity before computing the horizontal CoM trajectory.

### 2.2. Vertical CoM Generation

Without loss of generality, the motion of one periodic step was taken as an example to illustrate the planning of vertical CoM. A periodic step has two phases, the stance phase and the flight phase, as shown in Figure 1, and their duration is provided. The variation in the vertical CoM during the flight phase is determined by

(3) $$c^{z}\left(t\right)=z_{to}+{\dot{z}}_{to}t+\frac{1}{2}gt^{2},\;t\in \left[0,T_{fly}\right]$$

where $z_{to}$ and ${\dot{z}}_{to}$ are the height and rate of CoM at take-off (to), respectively, and $T_{fly}$ is the duration of the flight phase. For the sake of simplicity, the vertical velocity at take-off was set as the opposite to the ones at touchdown (td), i.e., ${\dot{z}}_{to}=-{\dot{z}}_{td}$. Then, ${\dot{z}}_{to}$ is given by

(4) $${\dot{z}}_{to}=\frac{gT_{fly}}{2}$$

Given the height, velocity, and acceleration of CoM at touchdown ($z_{td},{\dot{z}}_{td},{\ddot{z}}_{td}$) and take-off ($z_{to},{\dot{z}}_{to},{\ddot{z}}_{to}$), these quantities corresponded to ones at the beginning and end of the stance phase. With minimal squat height during stance $z_{low}$, the change in the vertical CoM is described by the following sextic polynomial:

(5) $$c^{z}\left(t\right)=\sum_{i=0}^{6}a_{i}t^{i},\;t\in \left[0,T_{sup}\right]$$

(6) $$\left\{\begin{array}{c} c^{z}\left(0\right)=z_{td},\;{\dot{c}}^{z}\left(0\right)={\dot{z}}_{td},\;{\ddot{c}}^{z}\left(0\right)={\ddot{z}}_{td}\\ c^{z}\left(T_{sup}/2\right)=z_{low}\\ c^{z}\left(T_{sup}\right)=z_{to},\;{\dot{c}}^{z}\left(T_{sup}\right)={\dot{z}}_{to},\;{\ddot{c}}^{z}\left(T_{sup}\right)={\ddot{z}}_{to} \end{array}\right.\;\Rightarrow a_{i}$$

where $T_{sup}$ is the duration of the stance phase, and $a_{i}\left(i\in \left\{0,1,2,\ldots ,6\right\}\right)$ are the coefficients of the sextic polynomial. $z_{low}$ is a manually adjustable quantity that mainly affects the vertical contact force profile during the stance phase.

### 2.3. Horizontal CoM Computation

After planning the desired periodic height trajectory of CoM, the horizontal CoM trajectory could be computed using (2) when the ZMP trajectory was provided, which was similar to [9,11]. However, it failed to satisfy the capturability constraint on the velocity of CoM during running. In this paper, ${\ddot{c}}^{x,y}$ in (2) was linearly approximated with the back Euler method as follows:

(7) $${\ddot{c}}_{i}^{x,y}=\frac{{\dot{c}}_{i}^{x,y}-{\dot{c}}_{i-1}^{x,y}}{dt}$$

where dt is the control period, and *i* represents the value of the *i*-th control period. Similarly, ${\dot{c}}_{i}^{x,y}$ and ${\dot{c}}_{i-1}^{x,y}$ can be approximated with the Euler method as follows:

(8) $${\dot{c}}_{i}^{x,y}=\frac{c_{i+1}^{x,y}-c_{i}^{x,y}}{dt},\;{\dot{c}}_{i-1}^{x,y}=\frac{c_{i}^{x,y}-c_{i-1}^{x,y}}{dt}$$

After (7) and (8) were plugged into (2), it was converted as follows:

(9) $$p_{i}^{x,y}=c_{i}^{x,y}-\frac{c_{i}^{z}}{\left({\ddot{c}}_{i}^{z}+g\right)dt^{2}}\left(c_{i+1}^{x,y}-2c_{i}^{x,y}+c_{i-1}^{x,y}\right)$$

Then, we could obtain the linear equation of ZMP and CoM at the *i*-th control period as follows:

(10) $$p_{i}=\left[\begin{array}{ccc} \alpha_{i} & \beta_{i} & \chi_{i} \end{array}\right]\left[\begin{array}{c} c_{i-1}\\ c_{i}\\ c_{i+1} \end{array}\right]$$

where x,y was omitted for simplicity, and $\alpha_{i},\beta_{i},\chi_{i}$ were dependent on the vertical CoM, $\alpha_{i}=\chi_{i}=-\frac{c_{i}^{z}}{\left({\ddot{c}}_{i}^{z}+g\right)dt^{2}}$ and $\beta_{i}=1-2\alpha_{i}$.

On the basis of (10), the relationship between ZMP and CoM for a limited horizon can be expressed as follows:

(11) $$\Phi c^{x,y}=p^{x,y}$$

where Φ is a matrix composed by $\alpha_{i},\beta_{i},\chi_{i}$.

The (11) does not contain any capturability constraints about CoM. Therefore, we modified it and add the capturability constraint on the velocity of horizontal CoM. The constraints are given by

(12) $$\left\{\begin{array}{c} c_{1}^{x,y}-c_{0}^{x,y}={\dot{c}}_{init}^{x,y}dt\\ c_{n}^{x,y}-c_{n-1}^{x,y}={\dot{c}}_{des}^{x,y}dt \end{array}\right.$$

where ${\dot{c}}_{init}^{x,y}$ and ${\dot{c}}_{des}^{x,y}$ are the initial CoM velocity at the beginning and desired CoM velocity at the end of the computing horizon, respectively.

The first and last lines of (11) were substituted with (12), and the resulting modified equation is as follows:

(13) $$\left[\begin{array}{ccccccccc} -1 & 1 & 0 & 0 & \cdots & 0 & 0 & 0 & 0\\ \alpha_{1} & \beta_{1} & \chi_{1} & 0 & \cdots & 0 & 0 & 0 & 0\\ 0 & \alpha_{2} & \beta_{2} & \chi_{2} & \cdots & 0 & 0 & 0 & 0\\ \vdots & \vdots & \vdots & \vdots & \ddots & \vdots & \vdots & \vdots & \vdots \\ 0 & 0 & 0 & 0 & \cdots & \alpha_{n-2} & \beta_{n-2} & \chi_{n-2} & 0\\ 0 & 0 & 0 & 0 & \cdots & 0 & \alpha_{n-1} & \beta_{n-1} & \chi_{n-1}\\ 0 & 0 & 0 & 0 & \cdots & 0 & 0 & -1 & 1 \end{array}\right]\left[\begin{array}{c} c_{0}\\ c_{1}\\ c_{2}\\ \vdots \\ c_{n-2}\\ c_{n-1}\\ c_{n} \end{array}\right]\;=\;\left[\begin{array}{c} {\dot{c}}_{init}dt\\ p_{1}\\ p_{2}\\ \vdots \\ p_{n-2}\\ p_{n-1}\\ {\dot{c}}_{des}dt \end{array}\right]$$

$p_{i},i\in \left\{1,2,\ldots ,n-1\right\}$ can be given as the center of the support polygon during the stance phase. When the robot is in the flight phase, it is not affected by any external force in the horizontal direction, so it moves at a constant velocity. Then, (10) can be written as follows:

(14) $$0=\left[\begin{array}{ccc} 1 & -2 & 1 \end{array}\right]\left[\begin{array}{c} c_{i-1}\\ c_{i}\\ c_{i+1} \end{array}\right]$$

Then, (14) was plugged into (13), which could be solved efficiently with the Thomas algorithm [27] at the computational cost of O(n).

## 3. Segmented ZMP Trajectory Optimization and Speed-Guided Foothold Iterative Computation

In this section, on the basis of the relationship between ZMP and the CoM trajectory (13) obtained in Section 2, we elaborate the strategies for smooth state switching and accurate tracking of the desired speed throughout the running.

### 3.1. Definition of Running State Switching

**Figure 2:**
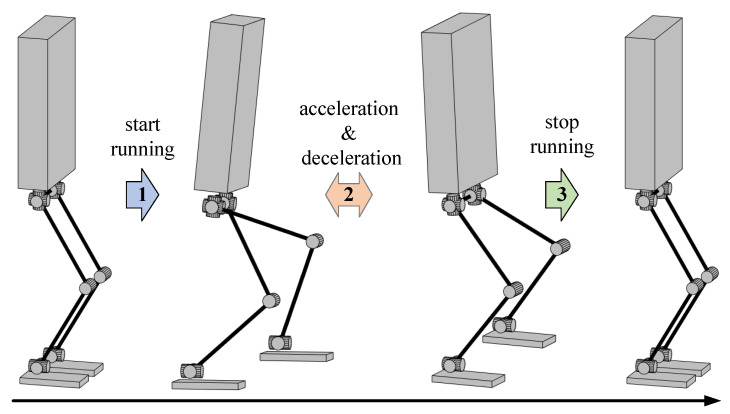
Switching of motion states during a complete run.

As shown in Figure 2, the complete running motion mainly involves three state switches between different motion states:

- State Switching 1: from bipedal support to cyclic single-leg support running at the beginning of running.
- State Switching 2: acceleration and deceleration during cyclic single-leg support running.
- State Switching 3: from cyclic single-leg support running to bipedal support at the end of running.

State Switching 1 and 3 can be classified into one category, and the difficulty lies in how to plan a feasible and smooth CoM trajectory to permit the robot to start or stop running stably at a given speed. The challenge of State Switching 2 is the online computation of the proper footholds to accurately track the desired speed during running.

### 3.2. Segmented ZMP Trajectory Optimization for Smooth State Switching

To tackle the difficulty of State Switching 1 and 3, we propose a segmented ZMP trajectory optimization formulation to plan the ZMP trajectory during bipedal support and realize the smooth transition of the CoM trajectory.

The following describes the formulation of the trajectory optimization with State Switching 1 as an example, and the problem of State Switching 3 is similar.

At the beginning of running, CoM movement is mainly in the lateral plane; for example, the robot requires to move its CoM to the supporting side to avoid falling towards the lifting side. From (13), it is possible to plan the CoM trajectory by providing the ZMP trajectory. As a consequence, a feasible CoM trajectory can be obtained by optimizing the ZMP trajectory to achieve smooth switching from stationary bipedal support to the periodic running state.

Assuming that the duration of bipedal support is given by *T*, the ZMP trajectory on the lateral plane is represented by y(t), and the boundary constraints of ZMP are provided by

(15) $$\begin{array}{c} y\left(0\right)=y_{0},\;\dot{y}\left(0\right)=0\\ y\left(T\right)=y_{1},\;\dot{y}\left(T\right)=0 \end{array}$$

where $y_{0}$ is the center of the bipedal support polygon, $y_{1}$ is the center of the single-leg support polygon, and $\dot{y}$ is the rate of ZMP that was set to 0 at the beginning and the end of bipedal support.

**Figure 3:**
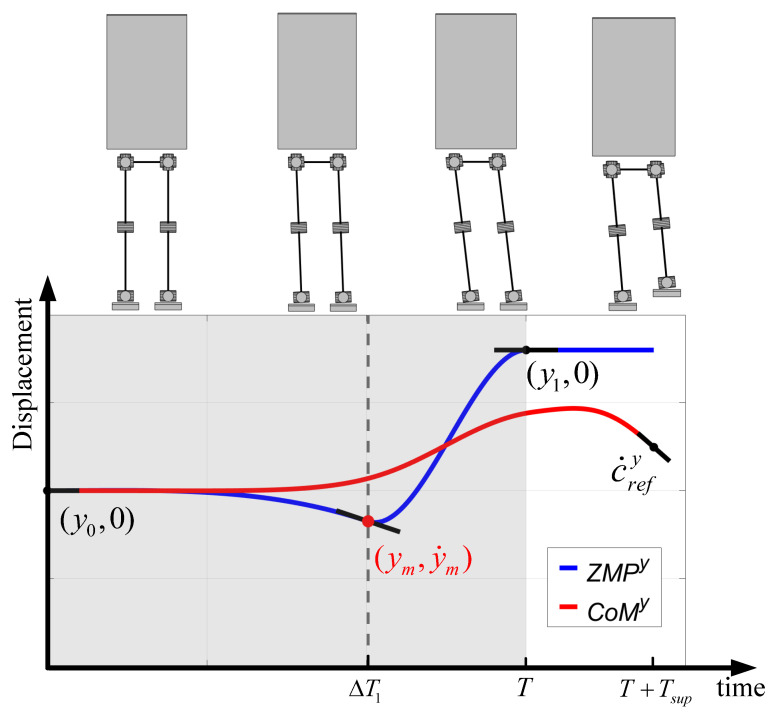
Segmented ZMP trajectory optimization during State Switching 1 (gray background). The short black lines are the tangent lines of two curves at the specified points. The robot switched from bipedal support to left-leg support.

In this paper, the ZMP trajectory during bipedal support was approximated with piecewise cubic polynomials, and the duration of each segment is represented by $\Delta T_{i}$(i∈{1,2,…,N}). For simplicity, N=2, as depicted in Figure 3. The ZMP and its rate at the knot point are denoted by $y_{m}$ and ${\dot{y}}_{m}$, respectively. Then, taking $\Delta T_{i}$, $y_{m}$, ${\dot{y}}_{m}$ as the decision variables, we could construct the following nonlinear programming problem:

$$\begin{array}{cc} find\; & \Delta T_{i},y_{m},{\dot{y}}_{m}\;\;\;i\in \left\{1,2\right\} \end{array}$$

(16a) $$\begin{array}{cc} s.t.\; & y\left(t\right)=\left\{\begin{array}{c} \sum_{j=0}^{3}b_{1,j}t^{j},\;t\in \left[0,\Delta T_{1}\right]\\ \begin{array}{c} \sum_{j=0}^{3}b_{2,j}{\left(t-\Delta T_{1}\right)}^{j},\;t\in \left(\Delta T_{1},T\right]\\ y_{1},\;t\in \left(T,T+T_{sup}\right] \end{array} \end{array}\right. \end{array}$$

(16b) $$\begin{array}{cc} \; & b_{1,j}=\Gamma \left(\Delta T_{1},y_{0},0,y_{m},{\dot{y}}_{m}\right) \end{array}$$

(16c) $$\begin{array}{cc} \; & b_{2,j}=\Gamma \left(\Delta T_{2},y_{m},{\dot{y}}_{m},y_{1},0\right) \end{array}$$

(16d) $$\begin{array}{cc} \; & \sum_{i=1}^{2}\Delta T_{i}=T \end{array}$$

(16e) $$\begin{array}{cc} \; & c^{y}\left(t\right)=\Phi {\left(c^{z},{\ddot{c}}^{z}\right)}^{-1}y\left(t\right),\;t\in \left[0,T+T_{sup}\right] \end{array}$$

(16f) $$\begin{array}{cc} \; & c^{y}\left(0\right)=c_{0}^{y},\;{\dot{c}}^{y}\left(0\right)=0,\;{\dot{c}}^{y}\left(T+T_{sup}\right)={\dot{c}}_{ref}^{y} \end{array}$$

(16g) $$\begin{array}{cc} \; & y(t)\in P,\;\forall t\in (0,T) \end{array}$$

We did not set an objective function for computational expediency, and the appropriate ZMP trajectory was considered found when the decision variables satisfied the above constraints. $T_{sup}$ was the predefined duration of single-leg support, and $b_{1,j}$ and $b_{2,j}$ were the cubic polynomial coefficients of two piecewise ZMP trajectories that were determined with Decision Variables (16b) and (16c). Equation (16d) constrained the total duration of bipedal support. Equation (16e) corresponds to (13) and was used to compute the CoM trajectory. The height of the CoM during bipedal support was constant for simplicity, and the variation in vertical CoM during the single-leg support is interpolated with (5) and (6). Equation (16f) enforced the boundary CoM states consistent with the desired states, and ${\dot{c}}_{ref}^{y}$ was the desired velocity of CoM at the end of the single-leg support. Equation (16g) restricted the planned ZMP during the bipedal support from exceeding the support polygon P. Γ() was the function to calculate the cubic polynomial coefficients given by

(17) $$b_{j}=\Gamma \left(\Delta T,x_{0},{\dot{x}}_{0},x_{1},{\dot{x}}_{1}\right),\;j\in \left\{0,1,2,3\right\}$$

(18) $$\left\{\begin{array}{c} b_{0}=x_{0}\\ b_{1}={\dot{x}}_{0}\\ b_{2}=-\frac{3x_{0}+2\Delta T{\dot{x}}_{0}-3x_{1}+2\Delta T{\dot{x}}_{1}}{\Delta T^{2}}\\ b_{3}=\frac{2x_{0}+\Delta T{\dot{x}}_{0}-2x_{1}+\Delta T{\dot{x}}_{1}}{\Delta T^{3}} \end{array}\right.$$

where ΔT is the duration of the cubic polynomial, $x_{0}$ and $x_{1}$ are the values of the end points, and ${\dot{x}}_{0}$ and ${\dot{x}}_{1}$ are their first derivatives, respectively.

Since there were only four decision variables in the constructed NLP problem (16), it could be solved efficiently online using off-the-shelf solvers such as SNOPT [28] and IPOPT [29] when the initial guesses are provided.

### 3.3. Iterative Online Speed-Guided Foothold Computation

In order to flexibly control the robot’s running speed online, we propose an iterative computation algorithm for the target footholds based on (13) with a CoM velocity capturability constraint to accurately track the commanded speed.

The iterative algorithm is illustrated in Algorithm 1. The robot entered the periodic running state after State Switching 1. For simplicity, we could treat the robot as oscillating periodically in the lateral plane, which allowed for concentrating on the following speed in the frontal plane.

The commanded translational speed ${\dot{c}}_{cmd}^{x,y}$ was communicated to the robot in the form of an online interaction (Line 1). Under the assumption that the step period was constant, the periodic vertical CoM trajectories could be easily generated using (5) and (6) (Line 2). Instead of predetermining the foothold of the next step, we provided a nominal foothold $p_{nom,j+1}^{x,y}$ that could be any point in the proximity of the current support foot (Line 3). The temporary CoM trajectory for the next step period $c_{temp,j+1}^{x,y}$ could be computed using (13) and the capturability constraints of the CoM velocity (Line 4). Comparing the errors of the CoM position $\Delta c^{i}$ at the transition, if the absolute value of the error was not greater than the tolerance (i.e., $tol=1\times 10^{-6}$), then the nominal foothold was the target foothold (Lines 7–8); if the absolute value of the error is greater than the tolerance, the CoM positional error is fed back into the temporary foothold and updates it (Lines 10–14). Line 4 is repeated until the absolute position error of the CoM is not greater than the tolerance. Then, the iteration computation was finished, and the temporary foothold of the last iteration was the target foothold (Lines 15–18). The target foothold obtained with this method not only enables the smooth switching of the CoM trajectory when the translational speed changes, but also accurately follows the desired speed at the take-off moment of the next step period.
**Algorithm 1** Iterative computation for target footholds1:Commanded translational speed: ${\dot{c}}_{cmd}^{x,y};$2:Using (5) and (6) to plan the vertical trajectory: $c_{j+1}^{z},{\ddot{c}}_{j+1}^{z};$3:Given nominal ZMP trajectory of next step: $p_{nom,j+1}^{x,y};$4:With (13) and capturability constraints of CoM velocity (${\dot{c}}_{j+1}^{x,y}\left(t_{0}\right)={\dot{c}}_{j}^{x,y}\left(t_{f}\right),{\dot{c}}_{j+1}^{x,y}\left(t_{f}\right)={\dot{c}}_{cmd}^{x,y}$), get temporary horizontal CoM trajectory: $c_{temp,j+1}^{x,y};$5:**for**$i\in \left\{x,y\right\}$**do**6:   $\Delta c^{i}=c_{temp,j+1}^{i}\left(t_{0}\right)-c_{j}^{i}\left(t_{f}\right);$7:   **if** $\left|\Delta c^{i}\right|\leq tol$ **then**8:     $\begin{array}{c} p_{tgt,j+1}^{i}=p_{nom,j+1}^{i};\;c_{tgt,j+1}^{i}=c_{temp,j+1}^{i}; \end{array}$9:   **else**10:     iter←0;11:     $p_{temp,j+1}^{i}=p_{nom,j+1}^{i};$12:     **while** $\left|\Delta c^{i}\right|>tol$ **do**13:        $p_{temp,j+1}^{i}=p_{temp,j+1}^{i}-\Delta c^{i};$14:        iter←iter+1;15:        Repeat Line 4 $\to c_{temp,j+1}^{i}$;16:        $\Delta c^{i}=c_{temp,j+1}^{i}\left(t_{0}\right)-c_{j}^{i}\left(t_{f}\right);$17:     **end while**18:     $\begin{array}{c} p_{tgt,j+1}^{i}=p_{temp,j+1}^{i};\;c_{tgt,j+1}^{i}=c_{temp,j+1}^{i}; \end{array}$19:   **end if**20:**end for**

**Figure 4:**
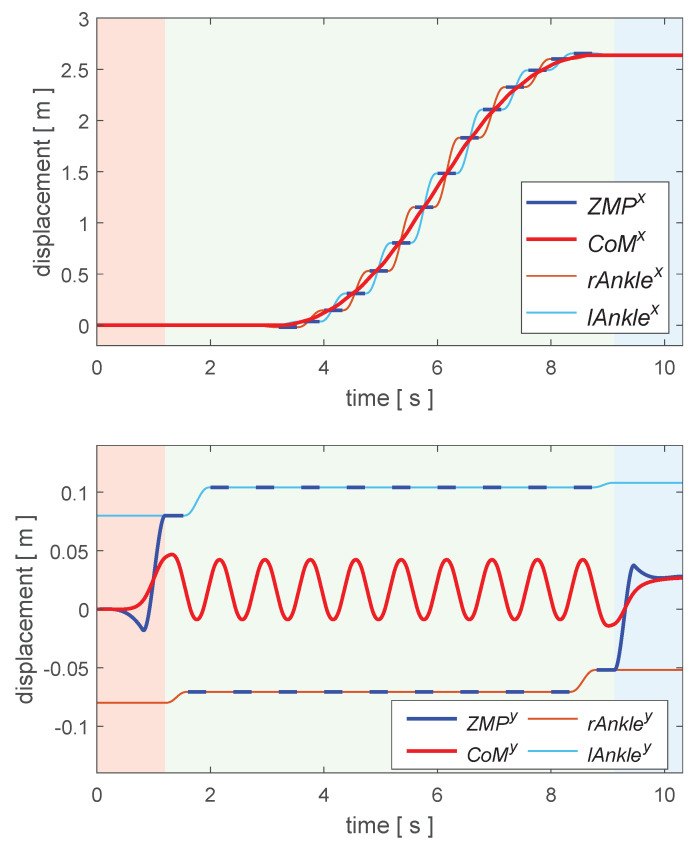
Trajectories of a complete running motion. Commanded forward speed increased first and then decreased, the maximal speed was 0.9 m/s, while the lateral speed changed periodically. The ZMP trajectories of State Switching 1 (red background) and 3 (blue background) were obtained with the NLP (16). The target footholds during the cyclic running (green background) were computed with Iterative Algorithm 1.

On the basis of the above running-gait generation method, interactively given the desired translational speed, it was efficient to obtain the footholds’ position and CoM trajectory as presented in Figure 4. The foot trajectory could be interpolated by sextic polynomials when the position of the footholds was calculated.

## 4. Results and Discussion

In this section, we outline how we used position-controlled bipedal robot BHR7P in combination with the proposed online running-gait generation method to verify its effectiveness in simulation. The results of the numerical experiment and online running simulation with the bipedal robot are visible in the accompanying video pink (video S1).

### 4.1. Robot Model and Simulation Setup

**Figure 5:**
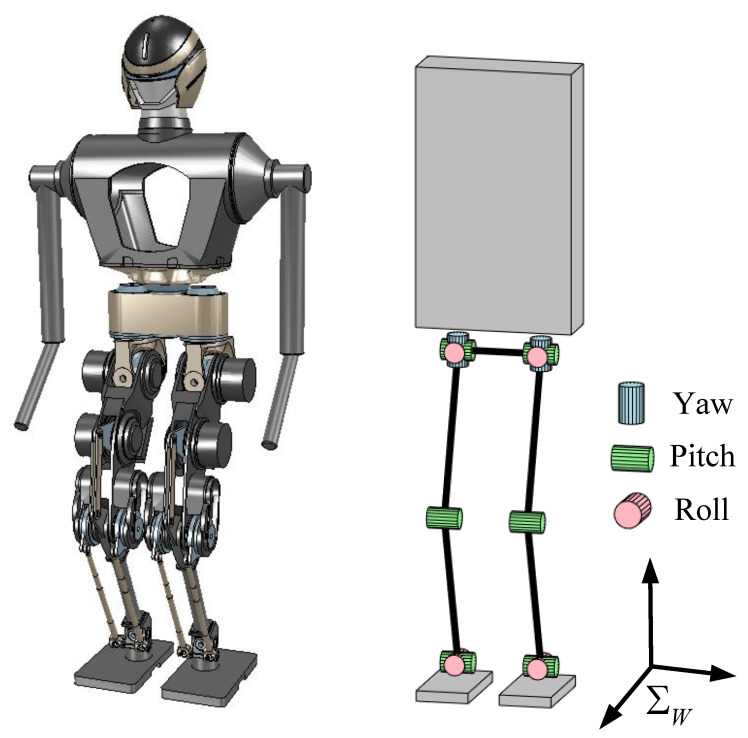
Bipedal robot BHR7P and its configuration.

Figure 5 shows that our position-controlled bipedal robot BHR7P [30] had 6 actuated degrees of freedom (DoFs) in each leg, comprising 3 DoFs at the hip (yaw, roll, pitch), 1 DoF at the knee (pitch), and 2 DoFs at the ankle (pitch, roll). The robot had a total weight of 55.9 kg and was 1.4 m high. The knee joint was actuated with a crank linkage mechanism, and the two ankle joints were driven by parallel differential linkage mechanisms. In the running motion planning, we approximated the CoM as the pelvic position.

The simulation experiments were implemented in CoppeliaSim software (V-rep) [31]. Taking the online planned running motion as the reference trajectory, we implemented the online balancing control strategy proposed in our previous work [32] during running with a control period of 4 ms.

### 4.2. Online Running Simulation

**Figure 6:**
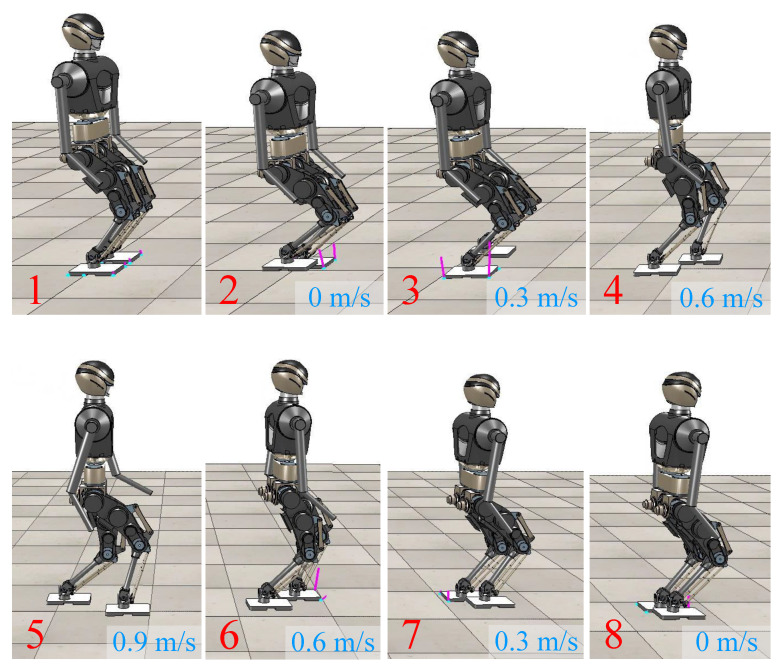
Snapshots of BHR7P running in simulation. The running speed of robot was controlled through online interaction.

As shown in Figure 6, we planned a complete running motion for BHR7P. The duration of the bipedal support was 1.2 s. The period of one step was 0.4 s, and the proportion of the stance phase was 0.7. It started from stationary and entered a cyclic running state. The running speed of the robot was controlled with a keyboard. When the robot received a new commanded speed, it arrived at the desired speed after the next two steps; otherwise, it kept the current movement speed. The target footholds during the cyclic running were derived with the iterative computation algorithm. The reason for achieving the desired speed after two steps was that the robot was alternately supported by two feet during running, which facilitated planning the trajectory of the swinging foot. Figure 6 shows that the robot’s forward speed was incremented or decremented by 0.3 m/s. There are remarkable flight phases during running, and the swing of the arms aims to balance the generated centroidal angular momentum.

**Figure 7:**
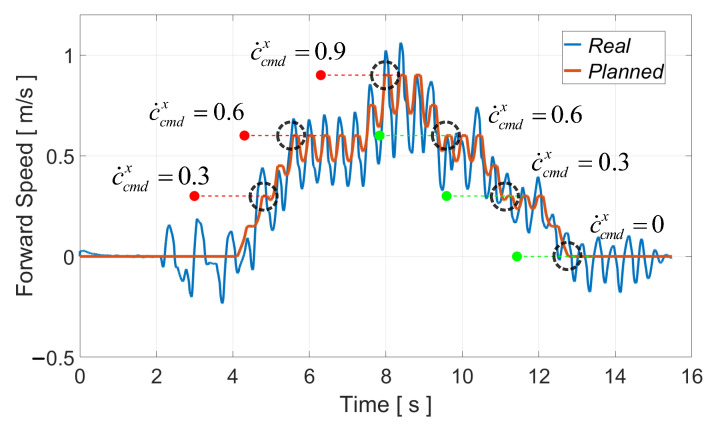
Real and planned forward speed during running.

Figure 7 presents the tracking of the commanded forward speed during running; the robot could follow the desired forward speed in the next two steps after finishing the current stride. The planned speed reached the commanded speed at the time of take-off, as illustrated in the black dotted circles in Figure 7, because the capturability constraint of the CoM velocity (12) was incorporated into running-gait generation. Considering the error of the dynamic model and sensor noise, it was acceptable that the actual estimated forward speed was slightly different from what was planned.

Regarding the online computational effort, the NLP (16) could be solved in less than 4 ms with IPOPT on a laptop with an Intel(R) Core(TM) i7-7700 CPU@3.60 GHz because there were only four decision variables and no objective function. The number of iterations to compute the target footholds for speed tracking is usually less than 10, and thanks to Linear Equation (13) of CoM and ZMP, the target footholds could be calculated within 1 ms. These enabled applying the proposed approaches to a real bipedal robot.

## 5. Conclusions and Future Work

In this paper, we proposed an online running-gait generator for bipedal robots with smooth state switching and accurate speed tracking. It could plan a complete and smooth running motion for the bipedal robot, and the running speed could be flexibly controlled via online interaction. Our conclusions are as follows.

- For smooth state switching at the beginning and end of running, segmented ZMP trajectory optimization was proposed to automatically plan a feasible and smooth CoM trajectory that enables the robot to start or stop running stably at the given speed.
- To accurately track the online interactive commanded speed, an iterative algorithm was designed to compute the target footholds with the capturability constraint of the CoM velocity, which allowed for the robot to flexibly accelerate or decelerate online, and enhanced its reactivity.
- Lastly, combined with the proposed approaches, bipedal robot BHR7P could accomplish a complete run with variable speed through online interaction in simulation, which demonstrated the validity of these approaches.

The limitation of this study is that current running-motion generation focuses on accurately following translational speed on flat ground. Speed tracking when a turning motion is required and the planning of running motion on nonflat terrain were not considered.

For future work, we will concentrate on implementing the proposed online running-motion planner on a real bipedal robot with balance control. In addition, we will investigate speed tracking for running turns and running motion planning on nonflat terrain.

## Data Availability

Not applicable.

## Supplementary Materials

The following supporting information can be downloaded at: https://www.mdpi.com/article/10.3390/biomimetics8010114/s1, Video S1: Numerical experiment and simulation of bipedal running.

## Abbreviations

The following abbreviations are used in this manuscript:

ZMP	Zero moment point
CoM	Center of mass
LIP	Linear inverted pendulum
VHIP	Variable-height inverted pendulum
TO	Trajectory optimization
NLP	Nonlinear programming
to	Take-off
td	touchdown
DoF	Degree of freedom

## References

[1] Kajita S., Kanehiro F., Kaneko K., Fujiwara K., Harada K., Yokoi K., Hirukawa H. Biped walking pattern generation by using preview control of zero-moment point. Proceedings of the 2003 IEEE International Conference on Robotics and Automation.

[2] Kuindersma S., Deits R., Fallon M., Valenzuela A., Dai H., Permenter F., Koolen T., Marion P., Tedrake R. (2016). Optimization-based locomotion planning, estimation, and control design for the atlas humanoid robot. Auton. Robots.

[3] Chen X., Yu Z., Zhang W., Zheng Y., Huang Q., Ming A. (2017). Bioinspired control of walking with toe-off, heel-strike, and disturbance rejection for a biped robot. IEEE Trans. Ind. Electron..

[4] Chen B., Zang X., Zhang Y., Gao L., Zhu Y., Zhao J. (2022). A non-flat terrain biped gait planner based on DIRCON. Biomimetics.

[5] Di Carlo J., Wensing P.M., Katz B., Bledt G., Kim S. Dynamic locomotion in the mit cheetah 3 through convex model-predictive control. Proceedings of the 2018 IEEE/RSJ International Conference on Intelligent Robots and Systems (IROS).

[6] Wang L., Meng L., Kang R., Liu B., Gu S., Zhang Z., Meng F., Ming A. (2022). Design and dynamic locomotion control of quadruped robot with perception-less terrain adaptation. Cyborg Bionic Syst..

[7] Ficht G., Behnke S. (2021). Bipedal humanoid hardware design: A technology review. Curr. Robot. Rep..

[8] Mikolajczyk T., Mikołajewska E., Al-Shuka H.F., Malinowski T., Kłodowski A., Pimenov D.Y., Paczkowski T., Hu F., Giasin K., Mikołajewski D. (2022). Recent advances in bipedal walking robots: Review of gait, drive, sensors and control systems. Sensors.

[9] Nagasaki T., Kajita S., Kaneko K., Yokoi K., Tanie K. A running experiment of humanoid biped. Proceedings of the 2004 IEEE/RSJ International Conference on Intelligent Robots and Systems (IROS).

[10] Tajima R., Honda D., Suga K. Fast running experiments involving a humanoid robot. Proceedings of the 2009 IEEE International Conference on Robotics and Automation.

[11] Takenaka T., Matsumoto T., Yoshiike T., Shirokura S. Real time motion generation and control for biped robot-2 nd report: Running gait pattern generation. Proceedings of the 2009 IEEE/RSJ International Conference on Intelligent Robots and Systems.

[12] Egle T., Englsberger J., Ott C. Analytical Center of Mass Trajectory Generation for Humanoid Walking and Running with Continuous Gait Transitions. Proceedings of the 2022 IEEE-RAS 21st International Conference on Humanoid Robots (Humanoids).

[13] Vukobratović M., Borovac B. (2004). Zero-moment point—Thirty five years of its life. Int. J. Humanoid Robot..

[14] Dai H., Valenzuela A., Tedrake R. Whole-body motion planning with centroidal dynamics and full kinematics. Proceedings of the 2014 IEEE-RAS International Conference on Humanoid Robots.

[15] Xin S., You Y., Zhou C., Tsagarakis N. Humanoid running based on centroidal dynamics and heuristic foot placement. Proceedings of the 2017 IEEE International Conference on Robotics and Biomimetics (ROBIO).

[16] Atlas (2021). Partners in Parkour.

[17] Sugihara T., Imanishi K., Yamamoto T., Caron S. 3D biped locomotion control including seamless transition between walking and running via 3D ZMP manipulation. Proceedings of the 2021 IEEE International Conference on Robotics and Automation (ICRA).

[18] Romualdi G., Dafarra S., L’Erario G., Sorrentino I., Traversaro S., Pucci D. Online non-linear centroidal mpc for humanoid robot locomotion with step adjustment. Proceedings of the 2022 International Conference on Robotics and Automation (ICRA).

[19] Orin D.E., Goswami A., Lee S.H. (2013). Centroidal dynamics of a humanoid robot. Auton. Robots.

[20] Mombaur K. (2009). Using optimization to create self-stable human-like running. Robotica.

[21] Grizzle J.W., Hurst J., Morris B., Park H.W., Sreenath K. MABEL, a new robotic bipedal walker and runner. Proceedings of the 2009 American Control Conference.

[22] Schultz G., Mombaur K. (2009). Modeling and optimal control of human-like running. IEEE/ASME Trans. Mechatron..

[23] Koolen T., De Boer T., Rebula J., Goswami A., Pratt J. (2012). Capturability-based analysis and control of legged locomotion, Part 1: Theory and application to three simple gait models. Int. J. Robot. Res..

[24] Pratt J., Koolen T., De Boer T., Rebula J., Cotton S., Carff J., Johnson M., Neuhaus P. (2012). Capturability-based analysis and control of legged locomotion, Part 2: Application to M2V2, a lower-body humanoid. Int. J. Robot. Res..

[25] Raibert M.H. (1986). Legged Robots That Balance.

[26] Caron S. Biped stabilization by linear feedback of the variable-height inverted pendulum model. Proceedings of the 2020 IEEE International Conference on Robotics and Automation (ICRA).

[27] Atkinson K.E., Han W. (1985). Elementary Numerical Analysis.

[28] Gill P.E., Murray W., Saunders M.A. (2005). SNOPT: An SQP algorithm for large-scale constrained optimization. SIAM Rev..

[29] Wächter A., Biegler L.T. (2006). On the implementation of an interior-point filter line-search algorithm for large-scale nonlinear programming. Math. Program..

[30] Li Q., Meng F., Yu Z., Chen X., Huang Q. (2021). Dynamic torso compliance control for standing and walking balance of position-controlled humanoid robots. IEEE/ASME Trans. Mechatron..

[31] Rohmer E., Singh S.P.N., Freese M. V-REP: A versatile and scalable robot simulation framework. Proceedings of the 2013 IEEE/RSJ International Conference on Intelligent Robots and Systems.

[32] Dong C., Chen X., Yu Z., Zhang Y., Chen H., Li Q., Huang Q. A unified control framework for high-dynamic motions of biped robots. Proceedings of the 2021 6th IEEE International Conference on Advanced Robotics and Mechatronics (ICARM).

